# The ‘Charter of Rights for Family Caregivers’. The role and importance of the caregiver: an Italian proposal

**DOI:** 10.1136/esmoopen-2017-000256

**Published:** 2017-11-04

**Authors:** Sisto Antonella, Vicinanza Flavia, Tartaglini Daniela, Tonini Giuseppe, Santini Daniele

**Affiliations:** 1 Department of Clinical Psychology, Campus Bio-Medico University Hospital of Rome, Rome, Italy; 2 Nursing Director, Campus Bio-Medico University Hospital of Rome, Rome, Italy; 3 Department of Medical Oncology, Campus Bio-Medico University of Rome, Rome, Italy

**Keywords:** caregiver, burden, cancer, psychological support.

## Abstract

Cancer diagnosis not only shakes the lives of those who are affected, but also has impacts on the entire family system, which is involved as if it were itself an organism affected by cancer. The oncological illness may cause a breakdown of the existing balance in the family system and demands a progressive degree of mutual adaptation to family members. The ‘VoiNoi’ Listening Centre of the Campus Bio-Medico University Hospital in Rome has been close to caregivers for several years, to support them in the difficult task of providing assistance through individual or group psychological support therapy and health education. The ‘Charter of Rights for Family Caregivers’ stems from the experience gained over the years, with the aim of protecting, supporting and strengthening the role and the assistance activity that families carry out in support of their loved ones under conditions of fragility.

**Key questions**

- In Europe, according to estimates, not self-sufficient patients receive 80% of the assistance from their spouse, children and other relatives. Caregiving involves a constant and long-term commitment, which can become overwhelming and cause high levels of stress and psychophysical discomfort.
- There is a need for care choices and professional support that allow families to experience the difficult task of caregiving, supporting and helping them dealing with the disease path to the best possible extent.
- The Charter of Caregivers’ Rights is the result of the experience gained over the years and it aims at recognising and enhancing the role of caregivers as a fundamental component of the care path. For caregivers, feeling recognised as bearers of rights, as well as providers of support to the patient, is a key step in favouring the activation of resilience processes.
- Recent literature emphasises the need to give importance to the role of the family in enhancing and supporting the person’s engagement in health management.
- Moreover, promoting family caregivers engagement in the care pathway is crucial to enhance the effectiveness of therapeutic intervention and to facilitate compliance with treatment. This Charter of Rights is part of an integrated approach to care, with the objective of supporting family caregivers effectively and innovatively, accompanying them in their heavy duty of helping the sick family member. In the literature, it emerges that increasing the engagement of caregivers and patients develops management behaviours, enhances awareness of the emotional dimension associated with the disease and promotes the quality of the relationship between cancer patients and their informal caregivers.

## Introduction

When a family member gets severely ill, this event may be experienced by his/her family as an unexpected, negative and often devastating one. In fact, the diagnosis of illness results in a breakdown of the pre-existing family balance and requires all members of the structure to mutually adapt to the new condition.^1^

The assistance and care pathway does not pertain only to the patient, but affects the entire family, often shaking their daily habits and social relationships.

In this context, the figure of the family caregiver plays a key role.

In literature, it is often noted that caregivers have prolonged exposure to stress factors called ‘burden’.^2^

This term actually contains a multidimensional concept and indicates the impact and negative consequences of the care burden on the psychophysical well-being and the quality of life of informal caregivers.^3^

The caregiver burden produces several repercussions, which may favour the onset of sleep disorders, depression, anxiety, asthenia, gastrointestinal disorders and headache, and even compromise the functioning of some daily life aspects, such as the personal space, the working role, relationships and family dynamics.^4 5^

In a study involving caregivers of patients with lung cancer, the 46.5% of the participants suffered from anxiety while depressed subjects were identified in the 27.9%. Moreover, the same study showed that the incidence of anxiety even exceeded cancer patients’ rate (46.5% vs 32.6%).^6^

This evidence has a great practical and operational value: it should not be forgotten that if caregivers get ill, instead of representing a resource, they may deteriorate a situation which is in and of itself already very difficult. It is therefore necessary for the healthcare staff to recognise the importance of caring and to timely intervene to prevent or reduce the onset of the caregiver burden.

Studies also show that as the family member’s disease progresses, the psychophysical problems related to the care burden tend to worsen, with direct consequences on the quality of life of caregivers.^7^

Literature suggested that caregivers’ quality of life could be influenced by many factors, such as their perception of the caregiving situation, their hopes and their ability of coping.

In many studies it has been demonstrated that the caregiver’ chance in quality of life can be associated with the patients’ cancer type. In particular, female-related cancers are usually associated with a poorer caregiver quality of life. In these cases, the caregivers are more likely to be men with difficulties coping with the patients’ cancer and dedicated themselves both to work and caregiving.^8^

It is therefore necessary to make choices in terms of care and professional support, so that families experiencing the difficult task of caregiving are duly supported, in order to face the disease in the best possible way.

## The Family Caregiver: an Italian proposal

Although caregivers are a population at risk, not all are ‘condemned’ to developing psychological distress. Their reactions are largely influenced by the coping and strain strategies they use, by the evaluation and meaning that they give to their experience and by their attitude to the disease.^9^ Considering the emotional dimension is important, too, every disease activates multiple emotional experiences, and both the patients and their family are called on to handle them. Again, the outcome is largely influenced by personal life stories, the coping and problem-solving strategies adopted to handle stress, the presence of a supporting social network or, conversely, the experienced social isolation.^10^

The ‘VoiNoi’ Listening Centre of the Campus Bio-Medico University Hospital in Rome represents a concrete answer to the needs of caregivers. This Centre is intended for family caregivers, with the aim of preventing and reducing the psychological distress conditions that they may develop in the care pathway of their loved family members.

To this end, a model has been developed to implement individual or group psychological support therapy aimed at reducing clinically significant distress situations.

Individual psychological support pathways identify and activate the caregivers’ resources to promote the process of family members adapting to the disease and to encourage their active and conscious participation in the care and assistance processes. The group of psychological therapy develops a support network around caregivers that promotes the encounter, exchange and sharing of thoughts, emotions and solutions among people who live the same suffering and face the same difficulties every day.^11^

The clinical activity is also carried out in collaboration with the Campus Bio-Medico Operational Units, in particular with the oncology unit. To this end, multidisciplinary team meetings with caregivers are planned in some phases of the disease: to communicate the diagnosis, explain and share treatment lines, and inform about the worsening of the clinical situation. Moreover, an orientation activity on healthcare regional services has been developed, in collaboration with the hospital social service, to help caregivers in their search for local services which may support them in assisting the sick person. Together with caregivers, the above-mentioned facility also analyses the social conditions in which patients and those who take care of them live, in order to offer a more comprehensive and efficient service.

Furthermore, a theoretical-practical training programme has been implemented (its fifth edition concluded recently), with the aim of delivering specific expertise in social healthcare and primary care at home. The training activity is intended to provide theoretical–practical skills about the care of sick people considering their primary needs and daily activities, by promoting the development of caregivers’ personal resources. Available data in literature show that the ability to acquire specific skills increases the levels of self-efficacy perceived in terms of care and self-care and—for caregivers—this is a protective factor against the onset of psychological distress and for the maintenance of a good quality of life.^12^

Hence, caregivers who know the disease and acquire the tools to manage daily difficulties and emergency situations are able to respond responsibly, effectively and safely to their assistance role.^13^

The training programme managed to enhance, in many ways, the caregiver’s quality of life by reducing emotional distress and improving emotional and functional well-being. Most of the patients (86.6%) and caregivers (80.6%) involved in the study agreed that the programme helped them cope with cancer. The majority of both patient and caregiver reported that they would recommended the programme to others going through the same experience. At the same time, the programme managed to improve the benefits gained from the cancer experience and the level of self-efficacy.^14^

The centre also carries out research and development activities to improve the quality of life of patients and their family caregivers.

Over the years, the centre has developed scientific research lines in the oncological context. Specific research protocols aim at evaluating the main symptoms of psychological discomfort of the pair patient and caregiver. Studies show that both patients with cancer and their family experience a state of fragility linked to the disease that exposes them to the onset of psychological discomfort and deteriorating quality of life.

Up to now, >1800 families have benefitted from the services provided by the VoiNoi Centre. The efficacy and quality of services was measured through the Service Satisfaction Scale-30^15^ on a sample of 80 families who constantly used the psychological support service for >3 months. Data show that >90% of them evaluate the quality of the offered services as excellent. In particular, the *Perceived Outcame-Efficacy* factor is ‘excellent’ for 93% of these families, while the *Doctor–Patient Transactions* are ‘excellent’ for 98% of the participants.

## The ‘Charter of Rights for Family Caregivers’

On the basis of the experience acquired over the years, the Campus Bio-Medico University Hospital of Rome adopted a ‘Charter of Rights for Family Caregivers’.

This Charter was conceived to highlight the main caregivers needs, which were identified through the ‘VoiNoi’ centre daily work and supported by data in the literature.

As a result, we know that informal caregivers needs are related to medical communication, in terms of higher quality and quantity of information. They reveal the need to be heard, to receive clear health information and more instructions for managing primary home care, and to be informed on the services where they can find resources and support.^16^

It is also necessary to legitimise the caregivers right to express their own emotional reactions and personal experiences related to the care of their loved ones, receiving specialist help where necessary to prevent or reduce the burden.^6^

In this regard, caregivers need their assistance-related distress to be recognised and legitimised, in order to acknowledge their own limits and abilities, work promptly on conditions of fatigue and psycho-physical stress and integrate the care burden with their own life.

For caregivers, finding their own dimension of life and recovering space for themselves must become an integral part of the care provided to the loved ones: in fact, this allows them to retain or find personal resources that can be applied to decision-making and assistance processes during the disease course.

Therefore, the* ‘Charter of Rights for Family Caregivers’* states that:

- Family caregivers have the right to receive appropriate information on the illness and proposed treatment, so that they can make informed decisions about the health of their family member, with the patient’s informed consent.
- Family caregivers are entitled to receive all necessary informations from the medical team, to assist their loved person, with the patient’s informed consent.
- Family caregivers have the right to obtain clear and exhaustive information in order to benefit from all regional services which may be useful in the care of their family member, with the patient’s informed consent.
- Family caregivers have the right to legitimise their feelings: it is normal for them to feel tired, sad, nervous or in difficulty during the care pathway.
- Family caregivers have the right to take care of themselves: ‘I must remember that all beautiful and pleasant things I can do for me will also positively influence my family member’.
- Family caregivers have the right to recognise their limits and ability: ‘I cannot expect to be able to do everything or to succeed in everything, to accept my limits means learning to discover my own resources’. Family caregivers have the right to maintain some personal living spaces: ‘As I do everything possible for my dear, so I must do it for me’.
- Family caregivers have the right to ask for and receive help: it is important to recognise their own needs and those of the dear one, learning to delegate.
- Family caregivers have the right to protect their own health: to have a healthy diet, to rest for an adequate number of hours and to undergo routine medical check-ups. This is not only a right but a duty, in order to properly handle the healthcare burden.
- Family caregivers have the right to access high-quality healthcare services, based on the definition and observance of precise standards.

The ‘Charter of Rights for Family Caregivers’ aims at helping informal caregivers in acknowledging the value of their role.

Thanks to this self-awareness. It will be easier for them to provide and request the necessary help in order to implement increasingly useful and effective coping and supporting strategies towards the loved ones.
